# 

**Published:** 1905-08-15

**Authors:** 


Vol. LIX. AUGUST 15, 1905. No. 8.

### THE DENTAL REGISTER

EDITED BY

NELVILLE S. HOFF, D.D.S.,

ANN ARBOR, MICH.

PRICE, ONE DOLLAR A YEAR IN ADVANCE.

Published Monthly by

SAMUEL A. CROCKER & CO.,

Ohio Dental and Surgical Depot.

35 to 39 W. 5th St., Cincinnati, O.

Entered at the Postoffice at Cincinnati O. as second-class mail matter

